# Associations Between Environmental Conditions and Executive Cognitive Functioning and Behavior During Late Childhood: A Pilot Study

**DOI:** 10.3389/fpsyg.2019.01263

**Published:** 2019-05-31

**Authors:** Diana H. Fishbein, Larry Michael, Charles Guthrie, Christine Carr, James Raymer

**Affiliations:** ^1^Department of Human Development and Family Studies, The Pennsylvania State University, University Park, PA, United States; ^2^RTI International, Research Triangle Park, NC, United States

**Keywords:** home environment, neighborhood conditions, cognition, behavior, development, late childhood

## Abstract

Numerous studies have established the influence of detrimental home conditions on child cognition and behavior; however, fewer have assessed these outcomes in the context of relatively “normal” range of home environmental conditions. Given the exquisite sensitivity to the environment of the neural substrates that undergird executive functioning (EF) and behavioral self-regulation in children, it is possible that a range of conditions within the home, even in the absence of maltreatment or economic deprivation, may impact these outcomes. The purpose of the present exploratory investigation was to further define the relationship between features of the home environment using the HOME inventory (a structured interview and observation of parent and child) and several dimensions of child EF and behavioral problems. In addition, this study sought to elucidate potentially differential associations between home and parent-reported neighborhood conditions—a hypothetically less direct influence on cognition in this age group—and level of child functioning. A battery of EF performance tasks and a widely-used checklist of behavioral problems were administered to 66 children, 8–11 years old from a lower middle income, working class sample. Results showed significant relationships between the home environment and several dimensions of EF and behavioral problems. In contrast, neighborhood conferred additional effects only on rule-breaking and aggression, not cognition, which is consistent with evidence that externalizing behavior in this age group becomes increasingly oriented toward outside influences. These findings warrant follow-up studies to establish causality. A broader program of research designed to delve further into the relationship between nuanced influences from the home and child cognition and behavior has implications for parenting strategies that foster healthy development. Neighborhood contexts should also be considered during early and mid-adolescent years based on existing studies and findings reported herein suggesting that this period of newfound autonomy and the heightened significance of peer relationships may influence externalizing behaviors, with implications for protective courses of action.

## Introduction

The present investigation addresses an aspect of the “ecobiodevelopmental” theoretical framework that views human behavior as emergent from the multifactorial interaction of a biological organism with its social and physical environment (Shonkoff, 2012). Advances in multiple fields point to an emerging model in which childhood experiences and exposures to socio-environmental factors directly affect the developing brain structure and function, which, in turn, affect one’s ability to self-regulate behavior and emotion. In that regard, the home environment is the single most profound influence on early child development in multiple domains of functioning (NCR/IOM, 2009; National Academies of Sciences, Engineering, and Medicine, 2016). In particular, a strong evidence-base has established an association between exposure to certain home childrearing conditions and basic cognitive functions and behavioral self-regulation (Caspi et al., 2000; Goodnight et al., 2012). The quality of the home childrearing environment includes features such as the level of family functioning, parenting approach, order (versus disorder) in the home, and enriching experiences to which children are exposed. These home environmental characteristics relate to the ways in which parents interact with their children, thereby exerting a significant impact on children’s overall development, including the instillation of social, cognitive and emotional regulatory skills needed for success in all domains of life. In the presence of unfavorable home conditions or dysfunctional relationships between children and caregivers, children are more likely to manifest poorly developed social skills, cognitive deficits, and behavioral problems (Wasserman et al., 1996; El Nokali et al., 2010; Byford et al., 2012).

Prevailing, more distal, neighborhood conditions have potential to further compound the harmful effects of deleterious home environmental conditions on child and adolescent behavior, or confer their own detrimental impacts (Kroneman et al., 2004; Kohen et al., 2008). Most profoundly, global conditions such as poverty, economic inequality, discrimination, poor housing conditions, and ill-equipped school systems directly and indirectly influence child development, health, and self-regulation of child behavior (Hackman and Farah, 2009; Ferguson et al., 2013; Elliott et al., 2016). Other interpersonal and individual level aspects of the external environment have also been associated with poor child development, including lack of supportive adults or “safety nets,” perceptions of danger and risky peer influences (Kroneman et al., 2004). Such conditions have been linked to social incompetencies, health problems and mental health disorders in both adults and children (Kenney, 2012). These associations may be mediated through direct exposure to adversities (e.g., community violence, poor medical care, and stressed parents) and the heightened perception of stress and fear.

Both of these proximal (home) and distal (neighborhood) environmental influences may increase liability for cognitive deficits and behavioral dysregulation (that often co-occur) via their influence on the developing brain (Caspi et al., 2000; NCR/IOM, 2009; Coley et al., 2013). A number of studies have established these relationships, particularly for children reared in low income or otherwise substandard conditions and for those enduring maltreatment (Brody et al., 2001; Coley et al., 2013). Given the implications for the general population, however, there is a need to more fully elucidate the influence of variability in home and neighborhood characteristics on indicators of child development under relatively “normal” conditions; e.g., average income range and no exposure to severe adversity or maltreatment. The focus of this line of research is largely on cognitive and self-regulatory behaviors (e.g., aggression, inattention, rule-breaking, anxiety) that are subserved by brain regions that are “experience-dependent” and, thus, exquisitely sensitive to environmental inputs throughout childhood and adolescence (Hackman and Farah, 2009).

The development and function of the prefrontal cortex (PFC) are particularly vulnerable to environmental inputs with potential for enduring impacts, for better or for worse. While positive, enriching, and supportive experiences can facilitate intact development of and functioning in the PFC, adverse experiences during this vulnerable developmental period may produce measurable and long-standing alterations in neurobiological systems that influence cognition and behavior. Of particular relevance are executive functions (EFs), higher-order cognitive skills modulated by the PFC, a few dimensions of which include problem solving, decision making, forethought, impulse control, working memory, and abstract reasoning. The development of EFs is a multistage process starting in early childhood when the building blocks for EF begin to form (Moriguchi et al., 2016; Breukelaar et al., 2017). The more complex features of EF, such as those listed above, are emergent in adolescence but do not coalesce until early adulthood as the PFC develops circuitry with lower structures in the limbic system that regulate emotion (Bava and Tapert, 2010). One important implication of this developmental process is that, until this neural circuitry reaches maturity, brain regions subserving EFs are highly susceptible to unfavorable experiences in the social environment, leading in turn to functional delays or deficits and poor self-regulation of behavior and emotions. What is less understood, however, is ways in which more subtle, “non-toxic” exposures to different types of parenting, home environment and neighborhood conditions impact these domains of functioning. And since the prevailing social environment is equally capable of positively altering biological processes, uncovering relationships between social influences and cognitive and behavioral processes that underlie children’s development has long-term utility for promoting successful outcomes.

Of significance, a home environment favorable to healthy child development has potential to mitigate some of the negative effects of these more macro-level conditions (e.g., neighborhood violence, lack of social supports, or disorder/decline) (Brookmeyer et al., 2005). In an important study, Odgers et al. (2012) found that maternal warmth and parental monitoring completely mitigated the effects of low income on children’s antisocial behavior. And Chung and Steinberg (2006) reported that parenting factors mediated the effects of structural and social characteristics of the neighborhood in serious juvenile offenders. Determining whether the broader neighborhood environment might confer some additional influence on developmental outcomes is important if we are to effectively and efficiently direct our precious preventive resources. For example, intervening at the level of the home environment to focus on types of parenting behavior and family functioning associated with protection against adverse outcomes may exert more powerful effects than concentrating on neighborhood conditions. It is also possible that neighborhood conditions exert a differential effect on certain cognitive and behavioral domains that also requires address. With increased understanding of their relative contributions to child development, there is potential to develop and implement a complement of interventions that is more specifically geared toward ameliorating influential and malleable conditions and improving trajectories of child behavior (Sandel et al., 2016; Minh et al., 2017).

Over the past decade, we are gaining a much fuller understanding of how far-reaching the full range of psychosocial influences are on cognitive functioning and behavior. While a significant literature base has established that cognitive and emotion regulatory functions and behavioral self-regulation can be adversely affected by severe and/or chronic adversity (e.g., child maltreatment, neglect, poverty) (McLaughlin et al., 2015; Kavanaugh et al., 2017), additional research is needed to flesh out the effects of a more typical range of family and social conditions (e.g., parental involvement, family functioning, social supports, and environmental enrichments) on these functional outcomes. In this preliminary investigation, we test hypotheses that, within a typical range, conditions in the home relate to the complex cognitive functions and behaviors of preadolescent children, accounting for effects of age, sex, and household income. EF dimensions selected for study have been shown in previous studies to be emergent during the age range in our sample and have been linked to risk for psychopathology (e.g., externalizing disorders). We further hypothesized that neighborhood conditions would not exert an additional influence on outcomes of interest over and above the home environment, with the expectation that proximal conditions would be relatively more impactful. As such, this study seeks to further establish and define the connection between aspects of the home environment and any additional effects from the broader neighborhood environment on EF and behavioral orientations.

## Materials and Methods

This research was designed as a cross-sectional study of children (8 to 11 years old) in counties in Indiana (Lake County) and Illinois (Will County, DuPage County). The study’s original aims were to discern the association between exposure to manganese and cognitive functioning and behavioral problems, with adjustments for the home and neighborhood environment, other metal exposures, and relevant covariates. The present investigation focused attention on the relationship between home and neighborhood environmental conditions and cognitive and behavioral functioning.

### Recruitment Strategy and Subject Selection

Lists of all housing units in the in-scope portions of DuPage and Will, as well as Lake Counties, as defined by Census block groups, were purchased from a commercial vendor, the Marketing Systems Group (MSG). The MSG list provided information that satisfied study criteria for participation of households; namely, whether or not they were expected to include at least one child between 8 and 10 years of age. Each household on the list was identified by a name, street address, and telephone number. To meet our recruitment goal, we also broadened our reach by posting flyers on Craig’s List and at other physical locations in our study areas.

Initially, approximately 1000 households across the study area received a letter briefly explaining the study and its significance, and alerting them that they would soon be contacted by a Research Associate (RA). RAs contacted households by telephone (using the purchased lists discussed above) to solicit participation and screen parents to determine eligibility, namely to determine (a) whether the house was built after 1930, and if built before 1930, whether there was peeling paint in the home, (b) the length of residence in the home, (c) whether the household included a child age 8–11 in the home, (d) whether the child had lived in the house for at least 5 years, and (e) whether the child had any severe physical or mental disorders that would make it difficult for him or her to respond to questions or play computer games designed to measure thinking processes. Families that lived in a home built before 1930 were excluded due to the likelihood of lead-based paint use (except for four homes where parents reported no lead paint or pipes were present). In addition, homes built between 1930 and 1975 whose residents reported any peeling paint inside were also excluded. See Table 1 for inclusion and exclusion criteria.

**Table 1:** Study participant exclusion/inclusion criteria.

Inclusion criteria	Exclusion criteria
• Child in the home between the ages of 8 and 10 • Male and female • All ethnic and racial groups • English speaking subjects will be included • Current residence in the same home • Has a parent or guardian willing to participate • Can provided written parental consent and child assent	• Known mental retardation, autism, psychosis, or other remarkable mental disorders that interfere with intellectual capabilities: the telephone script asks about disorders that would prevent a child from responding to questions or playing the computer games. • Lived in a home at any point built before 1930 (because of the possible presence of lead paint) • Living in a home built between 1930 and 1975 that have peeling paint inside • Participants who exclusively speak languages other than English • Residence(s) must have no lead paint or pipes

The 8–11 year old age group was selected as the optimal developmental period to study effects of neurotoxicity on childhood cognitive and behavioral skills. The developmental period encompassed here is characterized by increased demands for use of skills acquired earlier and is at the threshold for exhibiting dysregulated behaviors, given emerging autonomy and opportunities. Only one child per household was included to avoid overrepresentation from any given set of households. Interviewers asked for the ages of all children living in the household. If there was more than one child in the target age range, the interviewer selected the children who last had a birthday, irrespective of sex.

For eligible households, the study’s purposes and procedures were explained to a primary caregiver and a request made for a home visit to obtain written consent and assent for participation. The purpose of the study was presented as a test of the effects of possible exposure to manganese on motor, cognitive, and behavioral functions in children. During the home visit the RA interviewed the parent and the child together, as well as collected the biological and environmental samples. The final sample included 66 children, 8–11 years old (20 8-year-olds, 23 9-year-olds, and 23 10-year-olds). Twenty-nine of the children were female and 37 were male—50 had self-identified ethnicity as White or Hispanic, and the median yearly family income range was $63,000 with a range from $5000 to $176,000. See Table 2 for a description of the geographic region from which the sample was recruited.

**Table 2:** Sample characteristics.

•*N* = 66 ∘ 8 year olds = 20 ∘ 9 year olds = 23 ∘ 10 year olds = 23 • Parent age = mean: 41.35 (*SD* = 6.4) • Smoker in house = mean: 1.88 (*SD* = 0.33)	• Female = 29 • Ethnicity: White or Hispanic = 50 • Median yearly family income range = $63,000 ∘ Range from $5,000 to $276,000 • Child having experienced a serious illness = mean: 1.7 (*SD* = 0.46)

#### Informed Consent Procedures

RAs received the National Institutes of Health (NIH) human subjects research training protocols to manage distressed respondents and mandatory reporting issues, and intensive training on the interview, surveys, executive cognitive and behavioral measures, as well as the environmental and biological data collection procedures used in the study. During the home visit, the RAs obtained written parental informed consent for their own participation, written permission for their child to participate, and written informed assent from the child. The RTI International Institutional Review Board approved this study.

### Measures

Both caregivers and children completed various portions of the test battery. Child participants were evaluated using an IQ test [Wechsler Abbreviated Scale of Intelligence (WASI)] for descriptive purposes, and six EF tasks. A primary caregiver also completed a questionnaire about child behavioral and mood disturbances (e.g., antisocial behavior, depression, attention deficits, impulsivity, irritability, and aggression) and queried about home and neighborhood conditions, medical and behavioral history of the child, and other background information. All testing materials were developmentally appropriate, inoffensive, and relatively unobtrusive, taking no more than 1.5 h for both the youth and caregiver.

Two college educated (both had B.A.s) female research assistants were intensively trained in all aspects of the protocol, from human subjects research safety procedures to administration of the survey and cognitive battery. They were observed by a seasoned field supervisor who accompanied the RAs on a rotating and frequent basis.

Assessments and interviews were conducted in a quiet location in the child’s home to maintain confidentiality and privacy, and in ways that reduced distractions that would otherwise interfere with cognitive performance. Once the session was completed, participants received compensation (i.e., cash for parents and gift cards for the youth).

#### Demographic and Psychosocial Test Battery

A background questionnaire was completed by the caregiver to characterize the household in terms of income level, occupation of primary wage earner, family residential history, neighborhood and housing conditions, and environmental contaminants in the home (e.g., cigarette smoking, lead pipes, or paint to the extent this information was known to the residents). Data on the child was also captured, including age, grade level, and number of siblings. Also, parents were queried about pre- and peri-natal complications, prenatal drug exposure, medical history, head injury, and family history of psychological and behavioral disorders (e.g., ADHD, conduct disorder). (Note that there were only a few reports of these disorders; a larger study would be able to explore potential moderation by these factors.)

#### HOME Inventory

**FIGURE 1:**
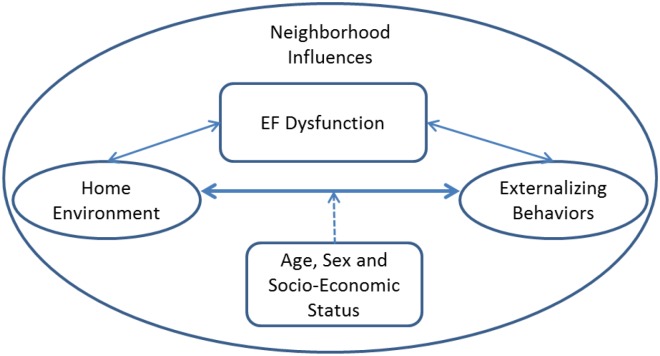
Conceptual model reflecting influence of home and family environments on externalizing behaviors via effects on EF, controlling for age, sex, and socio-economic status (SES).

Quality of the childrearing environment was measured by the HOME Inventory (Caldwell and Bradley, 2003). The HOME Inventory is a widely used semi-structured assessment that combines elements of interview and direct observation. It is composed of eight factor-analytically derived subscales with high interrater and test–retest reliability that assess the child-rearing qualities of the home. Scores have consistently been linked to child intelligence and achievement (Bradley et al., 1989; Molfese et al., 2003; Tong et al., 2007) and to disruptive psychopathology (Dubow and Ippolito, 1994; Pine et al., 1996; Wasserman et al., 1996; Wassermann, 1998). To reduce participant burden, we selected four subscales (Parent Responsivity, Emotional Climate, Enrichment and Family Companionship) chosen because of their high loadings in predicting children’s intelligence (Caldwell and Bradley, 2003) and, more importantly, based on our conceptual model which implicates these interpersonal features with externalizing behaviors (see Figure 1). The 33 items included in these subscales were summed to generate a HOME Total score.

#### Neighborhood Inventory

To evaluate neighborhood conditions, items were adapted from the National Study of Child and Adolescent Well-Being (NCSAW) survey (Bertolet et al., 2003). Caretakers were asked to rate the following items on a seven point Likert scale: graffiti on buildings and walls, abandoned buildings, abandoned cars, noisy, heavy traffic, speeding cars, crime, violence, drugs, trash, and litter. On a five point scale, caregivers were further asked if: they lived in a close-knit neighborhood, people know each other, the neighborhood is safe, is poorly maintained, is clean, has decent shopping areas, and has safe parks and playgrounds.

#### Cognitive Testing

Executive functioning abilities were measured in the child sample using non-invasive, developmentally appropriate, and specially designed cognitive tasks that have been related to PFC function. EF has multifactorial attributes that endure developmental changes over time and is supported by several subservient cognitive and emotional systems that are shaped earlier in childhood. Thus, EF measures selected are “preparatory” higher order cognitive skills that are prerequisite for full cognitive development as well as regulation of behavior and moods. Given reports that EF deficiencies are typified by a failure of behavioral inhibition, attention, working memory, consequence sensitivity, and problem solving, tasks that recruit brain regions which modulate these abilities (Kawashima et al., 1996; Casey et al., 1997; Konishi et al., 1998) were employed. These particular functions are also theoretically relevant to the extent to which an individual is at risk for behavioral dysregulation.

Executive functioning dimensions selected for this investigation were evaluated to identify specific deficits potentially associated with home and neighborhood conditions. The Cambridge Neuropsychological Test Automated Battery (CANTAB), used extensively in numerous studies of neurotoxic effects of various environmental conditions (e.g., both psychosocial and physical) on cognitive functions^1^, was employed to assess Spatial Working Memory (SWM between search errors), Rapid Visual Information Processing (RVP), Stockings of Cambridge (SOC), Information Sampling Task (IST) and the RTI simple reaction time (RTI simp). The tests included from the CANTAB have been extensively validated and normative data are available stratified by age from 4 to 80+ years old; we used the child’s version. Thus, we are able to compare the level of EFs in children with varying home and neighborhood conditions, controlling for income, sex, and race/ethnicity. See Table 3 for a description of the functions measured by each task.

**Table 3:** CANTAB cognitive task descriptions.

EF task	Function measured	Key variables
Rapid Visual Information Processing (RVP)	Visual Sustained Attention: detection of target sequences	A Prime: the signal detection measure of sensitivity to the target, regardless of response tendency.
Information Sampling Task (IST box)	Pre-decisional processing, where the subject gathers and evaluates information prior to making a decision	Mean boxes opened (box) and sample error.
Reaction Time (RTI simp)	Speed of response and movement in single paradigm	Simple paradigm.
Spatial Working Memory (SWM btw errors)	Working memory	Between search errors.
Stockings of Cambridge (SOC)	Spatial planning	Problems solved in minimum moves.

#### Behavior and Mood Assessment: Child Behavior Checklist (CBCL/6-18)

Certain Child Behavior Checklist (CBCL for 6–18 year olds) subscales were administered to the primary caretaker applying the standard method of scoring (Achenbach, 1991): Aggressive Behavior; Anxious/Depressed; Attention Problems; Rule-Breaking Behavior; Social Problems; Somatic Complaints; Thought Problems; and Withdrawn/Depressed. DSM-oriented scales include the following problem domains: Affective; Anxiety; Somatic; Attention Deficit/Hyperactivity; Oppositional Defiant; and Conduct Disorder. Reliability estimates range between 0.72 and 0.96 and external validity tested with age 12 CBCL t scores and other similar outcomes (e.g., Trauma Symptom Checklist and the Youth Self-Report Form) show significant (albeit modest) correlations ranging from 0.07 to 0.33 (Hunter et al., 2003a,b).

### Statistical Analyses

Initial exploratory and quantitative statistical analyses, as well as graphical presentations, were performed using SAS (v 9.3). Prior to quantitative analyses, frequencies, and summary statistics on categorical variables and continuous variables, respectively, were compiled. The total score computed from the four HOME subscales was included in models as a predictor. To consider associations with neighborhood conditions and EF and behavior, a new variable was created by summing the individual Neighborhood Inventory variables and scaling the result to a 0 to 1 scale, with “0” indicating no negative neighborhood conditions were reported.

Key cognitive variables from each CANTAB task were identified by selecting those that represent critical components of the function being measured, according to task parameters and the literature. Each behavioral scale and the total score on the CBCL were included in separate models. All analyses were performed using either SAS (v 9.3) or R (v 3.2.1). Descriptive statistical analyses were initially conducted to reveal missing data and extreme outliers which might adversely influence subsequent analyses—no changes were made to the dataset. Three step hierarchical multiple regressions were conducted with each cognitive and behavioral measure as the dependent variable to examine the degree to which the environmental variables predicted the cognitive and behavioral variables. Step one of the regression models included age, sex, and income to control for these potentially influential demographic factors. The main predictors of interest, total HOME score and neighborhood condition, were entered into the models at steps two and three, respectively, to determine whether differences emerged in associations between HOME and neighborhood conditions and cognitive/behavioral outcomes. An objective was to determine whether neighborhood characteristics explained additional variance for any outcomes over and above HOME scores.

## Results

A comprehensive suite of graphical and statistical diagnostics were performed on the data to ensure that the assumptions of linear modeling were upheld.

### Participant Score Distributions

Table 4 displays the analyses describing our sample, revealing that, in general, the total HOME score distribution was left-skewed and neighborhood condition score distribution was right-skewed, indicating that parenting conditions were perceived to be positive and nurturing, and the neighborhood environment was considered sufficient and safe, despite the lower income range of the sample. Behavior-related measures generally indicated that, in this sample, there were few reported behavior problems. Intelligence scores, SWM, IST, and the SOC solved in minimum moves measure showed overall normal distributions relative to the reference sample. The RVP distribution (toward the left) suggested an overall high level of sensitivity for detecting target sequences. Also, the RTI simp distribution (toward the right) showed that most participants responded relatively quickly in response to the onset of a stimulus in a single location (most RTs were under 332 ms). Z-scores for each measure indicate that participants performed within one standard deviation above or below normative scores from the general population.

### Environment and EF

At step one, the hierarchical regression models revealed that demographic characteristics, specifically age, contributed significantly to the SWM (between error) and RVP models—accounting for 17.7 to 23.0% (*p* < 0.01), respectively, of variation in these cognitive measures (see Table 5). When total HOME score was entered (i.e., step two), significant contributions were found in the RVP, RTI simp, IST box, and SOC models (p, with reports of more positive and nurturing home environments predicting better cognitive outcomes. Also, a more positive and nurturing home environment predicted higher WASI intelligence scores *R*^2^ = 0.16; *p* < 0.001). When neighborhood condition was added to the models (stage three), there were no changes: neighborhood did not contribute to any of the measured cognitive functions over and above relations with the home environment.

### Environment and Behavior

At step one, income was related to anxiety/depression and somatic symptoms (see Table 6). When total HOME score was entered into the regression models (step two), significant contributions were found in the total behavior, attention, and rule breaking models, indicating more positive and nurturing home environments were predictive of fewer reported behavioral issues. Neighborhood characteristics (step 3), on the other hand, did not confer any additional explanatory value in behavioral variations with the exception of rule breaking, showing a large increase (15.8%) in the context of poor neighborhood conditions. Interestingly, HOME score lost significance with neighborhood in the models. Thus, when demographic characteristics and home conditions are held constant, participants whose caregivers reported that their neighborhoods were clean and safe were reported to exhibit fewer rule breaking behavior issues. The Other Problem Behavior model did not show significant results at any stage of the regression analyses (*p* > 0.05).

## Discussion

The results of this investigation suggest that a relatively typical range of home conditions are measurably associated with levels of child EF and behavioral functioning in particular domains. Positive aspects of the home environment predicted better performance on two EF measures reflective of frontal lobe development, specifically visual information processing and working memory. It also predicted a lesser number of overall parent-reported behavioral issues. An advantage of the present investigation was the inclusion of a detailed quantitative examination of several EF dimensions in the context of usual variations in the quality of child rearing and parent-child relationships; the assessed set of experiences are not considered extreme or unusually harsh. And while we would expect a more nurturing environment to be associated with better behavioral performance, in the reverse, it appears from our findings that somewhat less favorable conditions may exert measurable negative impacts on child cognitive development.

**Table 4:** Summary statistics.

	Mean	*SD*	Median	Minimum	Maximum	Variable range
**Home environment**						
Total home scores	25.98	3.89	26.00	15.00	32.00	0–50
**Neighborhood condition**						
Total neighborhood condition	0.27	0.18	0.20	0.06	0.90	0.06–0.95
**Behaviors**						
Total behavior score	23.23	17.00	21.00	3.00	72.00	0–103
Aggressive behavior	4.52	4.47	3.00	0.00	18.00	0–18
Attention	3.55	3.55	3.00	0.00	15.00	0–15
Anxiety/depression	3.00	2.52	2.00	0.00	10.00	0–13
Rule breaking behavior	1.80	1.91	1.00	0.00	9.00	0–17
Somatic behavior	1.82	2.54	1.00	0.00	12.00	0–12
**Cognitive functioning**						Z-scores
RVP mean A prime	0.94	0.06	0.96	0.75	1.00	−0.396
IST sample error	2.20	1.45	2.00	0.00	5.00	No norms
RTI simple reaction time	332.36	67.11	320.80	231.50	604.38	0.565
SWM between search errors	42.30	16.53	45.00	11.00	75.00	−0.232
SOC solved in minimum moves	6.54	2.04	7.00	0.00	11.00	−0.586
IQ	105.71	13.25	106.50	73.00	129.00	Mean = 100

*SWM, Spatial Working Memory; RVP, Rapid Visual Information Processing; SOC, Stockings of Cambridge; IST, Information Sampling Task; RTI, the RTI.*

**Table 5:** Summary of hierarchical regressions for variables predicting EF measures.

		Step 1			Step 2			Step 3		
			CI 95%			CI 95%			CI 95%	
	Predictors	*B*	LL	UL	*B*	LL	UL	B (β)	LL	UL
IQ	Age	2.605	−1.889	7.099	3.411	−0.907	7.729	3.33 (0.17)	−1.023	7.691
	Sex (M)	−0.843	−8.100	6.414	−0.896	−7.798	6.005	−1.4 (−0.03)	−8.611	5.793
	Income (> PL)	1.183	−6.014	8.380	2.882	−4.084	9.848	2.97 (0.10)	−4.052	9.991
	Home				1.211^∗^	0.291	2.131	1.08^∗^ (0.35)	0.038	2.124
	Neighborhood							−6.13 (−0.004)	−28.747	16.492
	*R*^2^	0.028			0.137			0.142		
	*F*	0.535			6.869^∗^			0.295		
RVP	Age	0.035^∗∗^	0.017	0.053	0.038^∗∗^	0.020	0.055	0.04^∗∗^ (0.03)	0.019	0.054
	Sex (M)	−0.001	−0.030	0.028	−0.002	−0.030	0.027	−0.01 (−0.19)	−0.035	0.023
	Income (>PL)	−0.004	−0.033	0.025	0.002	−0.027	0.030	0.00 (0.13)	−0.026	0.031
	Home				0.004^∗^	0.000	0.008	0.00 (0.47)	−0.002	0.007
	Neighborhood							−0.06 (−0.42)	−0.148	0.035
	*R*^2^	0.220			0.277			0.297		
	*F*	5.263^∗^			4.366^∗^			1.534		
RTI simp	Age	−6.911	−30.589	16.767	−2.432	−25.327	20.463	−2.35 (−0.06)	−25.502	20.812
	Sex (M)	−11.584	−49.875	26.708	−12.590	−49.172	23.991	−11.95 (−0.06)	−50.244	26.355
	Income (>PL)	0.602	−37.279	38.483	8.513	−28.212	45.239	8.41 (0.18)	−28.700	45.527
	Home				6.094^∗^	1.242	10.947	6.25^∗^ (0.32)	0.753	11.755
	Neighborhood							7.58 (0.09)	−111.388	126.557
	*R*^2^	0.012			0.116			0.116		
	*F*	0.218			6.226^∗^			0.016		
SWM (btw)	Age	−9.865^∗∗^	−14.910	−4.820	−10.47^∗∗^	−15.485	−5.461	−10.4^∗∗^ (−0.02)	−15.442	−5.327
	Sex (M)	0.747	−7.399	8.893	0.787	−7.224	8.798	1.38 (0.11)	−6.979	9.741
	Income (>PL)	−0.936	−9.015	7.144	−2.219	−10.305	5.867	−2.32 (−0.02)	−10.470	5.830
	Home				−0.915	−1.983	0.153	−0.76 (−0.54)	−1.975	0.447
	Neighborhood							7.10 (0.25)	−19.155	33.357
	*R*^2^	0.221			0.261			0.265		
	*F*	5.297^∗^			2.907			0.294		
IST box	Age	−2.260	−4.481	−0.038	−2.620^∗^	−4.779	−0.462	−2.50^∗^ (−0.01)	−4.613	−0.376
	Sex (M)	−0.898	−4.485	2.689	−0.874	−4.324	2.576	−0.01 (−0.12)	−3.533	3.471
	Income (>PL)	0.564	−2.993	4.122	−0.196	−3.678	3.286	−0.34 (−0.21)	−3.754	3.074
	Home				−0.542^∗^	−1.002	−0.082	−0.33 (−0.23)	−0.836	0.179
	Neighborhood							10.08 (0.20)	−0.915	21.082
	*R*^2^	0.073			0.158			0.208		
	*F*	1.470			5.823^∗^			3.379		
SOC	Age	0.202	−0.492	0.896	0.410	−0.245	1.065	0.36 (0.01)	−0.286	0.999
	Sex (M)	−0.681	−1.816	0.454	−0.783	−1.834	0.268	−1.05 (−0.07)	−2.112	0.017
	Income (>PL)	−0.835	−1.964	0.294	−0.508	−1.571	0.556	−0.48 (−0.06)	−1.519	0.560
	Home				0.234^∗^	0.086	0.382	0.17^∗^ (0.11)	0.005	0.328
	Neighborhood							−3.43 (−0.09)	−7.094	0.231
	*R*^2^	0.068			0.220			0.270		
	*F*	1.283			10.613^∗^			3.538		

*^∗^p < 0.05; ^∗∗^p < 0.01. Sex (M) = male. Income (>PL) = over the poverty line. Standardized betas are provided for full models.*

**Table 6:** Summary of hierarchical regressions for variables predicting behavioral measures.

		Step 1			Step 2			Step 3		
			CI 95%			CI 95%			CI 95%	
	Predictors	*B*	LL	UL	*B*	LL	UL	*B* (β)	LL	UL
Total behavior	Age	1.495	−3.966	6.957	0.387	−4.766	5.541	0.72 (0.15)	−4.311	5.744
	Sex (M)	2.673	−6.145	11.492	2.747	−5.491	10.984	4.95 (0.04)	−3.366	13.256
	Income (>PL)	5.556	−3.190	14.303	3.221	−5.093	11.535	2.85 (0.34)	−5.257	10.948
	Home				−1.665^∗∗^	−2.763	−0.567	−1.11 (−0.05)	−2.311	0.096
	Neighborhood							26.30 (0.23)	0.199	52.404
	*R*^2^	0.035			0.173			0.231		
	*F*	0.669			9.750^∗∗^			4.081		
Anxiety/depression	Age	−0.029	−0.842	0.785	0.037	−0.784	0.857	0.04 (0.01)	−0.795	0.864
	Sex (M)	0.844	−0.470	2.157	0.839	−0.472	2.150	0.83 (0.14)	−0.546	2.197
	Income (>PL)	1.378^∗^	0.075	2.681	1.516^∗^	0.193	2.839	1.52^∗^ (0.12)	0.181	2.856
	Home				0.098	−0.076	0.273	0.10 (0.15)	−0.104	0.293
	Neighborhood							−0.17 (−0.02)	−4.476	4.141
	*R*^2^	0.092			0.112			0.112		
	*F*	1.887			1.250			0.006		
Somatic	Age	−0.393	−1.191	0.405	−0.459	−1.263	0.345	−0.43 (−0.19)	−1.232	0.375
	Sex (M)	−0.764	−2.052	0.525	−0.759	−2.045	0.526	−0.56 (−0.11)	−1.886	0.771
	Income (>PL)	1.412^∗^	0.134	2.690	1.273	−0.024	2.570	1.24 (0.05)	−0.057	2.534
	Home				−0.099	−0.270	0.072	−0.05 (−0.08)	−0.240	0.144
	Neighborhood							2.41 (0.23)	−1.760	6.584
	*R*^2^	0.124			0.145			0.165		
	*F*	2.636			1.350			1.343		
Attention	Age	0.489	−0.690	1.667	0.164	−0.866	1.193	0.22 (0.05)	−0.804	1.234
	Sex (M)	1.506	−0.397	3.409	1.527	−0.119	3.173	1.87^∗^ (0.20)	0.185	3.555
	Income (>PL)	0.337	−1.550	2.225	−0.348	−2.010	1.313	−0.41 (0.17)	−2.050	1.236
	Home				−0.489^∗∗^	−0.708	−0.269	−0.4^∗∗^ (−0.5)	−0.646	−0.158
	Neighborhood							4.12 (0.20)	−1.186	9.398
	*R*^2^	0.049			0.302			0.33		
	*F*	0.971			20.425^∗∗^			2.419		
Rule breaking	Age	−0.174	−0.788	0.440	−0.322	−0.881	0.238	−0.26 (−0.05)	−0.767	0.245
	Sex (M)	−0.344	−1.336	0.647	−0.335	−1.228	0.559	0.07 (0.02)	−0.768	0.905
	Income (>PL)	0.060	−0.923	1.044	−0.251	−1.154	0.651	−0.32 (−0.41)	−1.136	0.495
	Home				−0.222^∗∗^	−0.341	−0.103	−0.12 (−0.07)	−0.241	0.001
	Neighborhood							4.82^∗∗^ (0.29)	2.197	7.450
	*R*^2^	0.013			0.213			0.371		
	*F*	0.254			17.138^∗∗^			13.557^∗∗^		
Aggression	Age	0.066	−1.387	1.519	−0.139	−1.569	1.291	−0.05 (−0.05)	−1.444	1.347
	Sex (M)	0.081	−2.265	2.428	0.095	−2.190	2.380	0.70 (0.06)	−1.607	3.007
	Income (>PL)	0.323	−2.004	2.651	−0.108	−2.415	2.198	−0.21 (−0.05)	−2.461	2.037
	Home				−0.308	−0.612	−0.003	−0.16 (−0.17)	−0.489	0.180
	Neighborhood							7.24 (0.32)	−0.008	14.484
	*R*^2^	0.001			0.071			0.135		
	*F*	0.028			4.325^∗^			4.011		

*^∗^p < 0.05; ^∗∗^p < 0.01. Sex (M) = male. Income (>PL) = over the poverty line. Standardized betas are provided for full models.*

The emergence of EFs throughout childhood and adolescence is a dynamic, “experience-dependent” process, which translates to the exquisite sensitivity of brain development and function to environmental exposures. The literature is replete with studies documenting the impact of early adversity (e.g., child maltreatment, poverty, witnessing violence) on neurocognitive development throughout childhood and adolescence and, in turn, how adversity-related deficits or delays in neurocognitive function in youth can increase vulnerability to a myriad of maladaptive behaviors, both internalizing and externalizing (Toth and Cicchetti, 2013). Integrity of neurocognitive development translates to the ability to self-regulate behavior and emotion via “top down” cognitive control over affective responses to life’s challenges. The development of these processes may be particularly influential in adaptations to adversity. Thus, variations in neurocognitive trajectories are likely to be more pronounced in populations where adversity prevails which, in turn, may correspond to a wide range of behavioral pathways and outcomes, from low to high risk.

In contrast, findings from the present study provide support (albeit correlational) for a growing knowledge base suggesting that even a normal range of early experiences may more subtly impact neurobehavioral development. It is possible that even mildly negative conditions within the home have potential to compromise cognitive functioning in subtle ways, with directionality depending upon the nature of the influence (Glaser, 2000; McCrory et al., 2010; Sarsour et al., 2011; McEwen and Morrison, 2013). Sarsour et al. (2011), for example, found low SES to be negatively related to several dimensions of EF and that the home environment mediated this relationship. These results are similar to those reported herein, however, in the present study, controls were in place for income, suggesting a more direct effect of features of the proximal environment irrespective of SES. We surmise that intact executive functioning may mitigate the effects of a less than optimal home environment on behavioral outcomes; however, fully exploring this question requires a temporal component, which is out of the purview of this study due to the small sample size and lack of longitudinality. Future studies that are sufficiently powered to evaluate mediation will enable us to better understand ways in which social experiences impact and interact with EF to predict behavioral outcomes. Developing more precision-based interventions relies on a clearer delineation of critical time points when factors that are influential in maladaptive behavior, such as home environment, act on emergent neurocognitive systems in a manner that increases the likelihood of following one behavioral pathway versus another. Missing these time-dependent opportunities to intervene and redirect development may translate to higher risk for behavioral problems.

Interestingly, when neighborhood conditions were considered over and above the home environment, there was no effect on the dimensions of EF measured and little effect on most behavioral constructs. The two associations that withstood adjustments for covariates and home environment were between rule-breaking and aggression with neighborhood conditions. Brody et al. (2001) found associations between deviant peer relationships and both parenting and neighborhood conditions, suggesting that during the age range in the present study, acting out behavior outside the home (i.e., rule breaking) may be in part a function of exposure to deviant peers; unfortunately, in the present study, we did not include peer measures to explore this possibility in our sample. Chung and Steinberg (2006) also reported neighborhood conditions to be related to delinquency, which may manifest at earlier ages as rule-breaking and aggression. The lack of association with EFs suggest that neighborhoods may impact behavior through alternative routes when children become more autonomous, have greater separation from parents, and rely increasingly on friendship groups for their developing behavioral repertoires.

Important to consider in interpreting our findings is that the home and the neighborhood environments are indexed by quite different features, with HOME subscales focusing on social and relational qualities and the neighborhood measure focusing on physical conditions (e.g., graffiti, traffic, poorly maintained buildings). There are likely physical characteristics of the home environment (e.g., high level of clutter, lack of hygiene, unsafe conditions) that also influence child outcomes, as well as relational aspects of the neighborhood that were not considered in this study. Interestingly, a large number of recent studies are highlighting the influence of physical qualities of life environment for cognitive and attentional behavior (including variables also considered in the present study) in children and adolescents. And some studies report that the environment may have restorative properties in children, such as improvements in social behavior, attention, mood, and stress management (Carrus et al., 2015; Collado and Staats, 2016; Schutte et al., 2017; van den Berg et al., 2017). Such evidence suggests that other qualities of the developing child’s ecological surroundings (e.g., outdoor venues or contact with nature during school hours), not measured herein, may exert beneficial effects and, although, not yet studied, there may be potential to partially mitigate the impacts of more proximally experienced stressful events on cognition and behavior.

Conclusions from the present investigation’s findings are constrained in several respects. There is a possibility that other more severe conditions in the home and neighborhood environments may correspond with the more readily observable and reportable conditions that so highly relate to our cognitive and behavioral outcomes. For example, the combined low levels of enrichment, responsivity, family companionship and nurturing emotional climate from the HOME interview may be proxies for underlying abuse or neglect. Such questions built into the HOME inventory are reflective of these more severe experiences; thus, the conditions within the home can viewed on a continuum that may be inclusive of these harsher scenarios. Nevertheless, subsequent studies should incorporate other more objective measures of both home and neighborhood conditions and not rely solely on parental reports.

Other more obvious shortcomings pertain to the small sample size in this pilot study. Nevertheless, the relations are consistent in their directionality and reflect reasonably strong associations that are not likely to diminish with additional subjects. Further research with a larger and more representative sample will help to further specify these potentially differential associations. And as mentioned above, longitudinal studies are needed to flesh out temporality and the possibility that alterations in EF may mediate susceptibility to behavioral problems.

The home and neighborhood assessments employed in this study are affordable and readily administrable, thus having potential to be employed in various settings to identify home and neighborhood conditions that increase risk for cognitive and behavioral dysregulation that are in need of intervention. The protocol used herein, once replicated, may inform the development of screening tools that could be administered to subgroups at particularly high risk for developmental problems, such as in high poverty households, juvenile detention facilities, alternative schools and/or clinical populations for interventions that target specific aspects of the environment. Results of assessments, for example, may help to direct practitioners toward appropriately targeted prevention programs, cognitive rehabilitation approaches and communities in need of additional social supports for children and their families. Perhaps most compelling is that this information may help to guide public educational campaigns regarding conditions that are conducive to healthy development and, conversely, those that have potential to compromise the ability of children to reach their potential.

## Conclusion

The significance of this overall line of research is several-fold. First, elucidating neurobehavioral effects of the home environment can lead to a more sensitive and detailed ascertainment than surveys alone; thus, there is a greater likely of identifying deficits and delays that often become compounded over time. When these impairments are not recognized, interventions are either not implemented or not appropriately targeted, increasing risk for ongoing difficulties in self-regulation, learning, and cognitive control over emotional responses. Second, the potential for a nurturing home environment to partially mitigate some of the untoward effects of more adverse conditions, such as poverty or maltreatment, on child development would suggest that parenting and family interventions may hold great promise. A seminal study by Brody et al. (2016) provides support for this speculation, reporting that family intervention can significantly improve brain function in high poverty children. And third, evidence for the veracity of this framework has implications for social welfare and public health policies that focus on environments capable of optimizing child development. Of relevance to all these points, investigations by a Harvard group to study classroom intervention/cognitive training to support executive functioning show that benefits were especially strong among youth from disadvantaged economic backgrounds^2^. Although many environment-based interventions (home, school, community) are emerging that support healthy child development, we need better understanding of differential associations in these individual domains before we can employ a more effective approach to improve cognitive function and self-regulation.

## Ethics Statement

This study was carried out in accordance with the recommendations of “name of guidelines, name of committee” with written informed consent from all subjects. All subjects gave written informed consent in accordance with the Declaration of Helsinki. The protocol was approved by the Institutional Review Board of the RTI International.

## Author Contributions

DF conceived of and initiated this investigation, designed the test battery, provided oversight of the training and activities of data collectors including ensuring adherence to human subjects research procedures, assisted in data analysis, monitored expenditures, coordinated with collaborators, and wrote approximately 75% of the manuscript. LM played a role in the conception and design of the study, as well as conducted data analysis, contributed to the writing, and edited drafts. CG led the data analysis and wrote initial drafts of the statistical and results sections. CC trained and supervised the data collectors, problem solved when necessary, ensured compliance with human subjects research procedures, and processed data to produce an analyzable dataset. JR contributed significantly to the conception of the study, provided scientific and practical guidance throughout the investigation, and reviewed drafts of the manuscript.

## Conflict of Interest Statement

The authors declare that the research was conducted in the absence of any commercial or financial relationships that could be construed as a potential conflict of interest.

## Footnotes

1 http://www.cantabeclipse.co.uk/science/bibliography.asp

2 http://www.nsf.gov/discoveries/disc_summ.jsp?=y&cntn_id=132292&org=NSF
